# In memory of Professor Henry Wilde, MD, FACP: infectious disease physician, clinical and public health investigator, and educator

**DOI:** 10.2478/abm-2024-0013

**Published:** 2024-06-28

**Authors:** Editorial Office of Asian Biomedicine

**Affiliations:** Faculty of Medicine, Chulalongkorn University, Bangkok 10330, Thailand


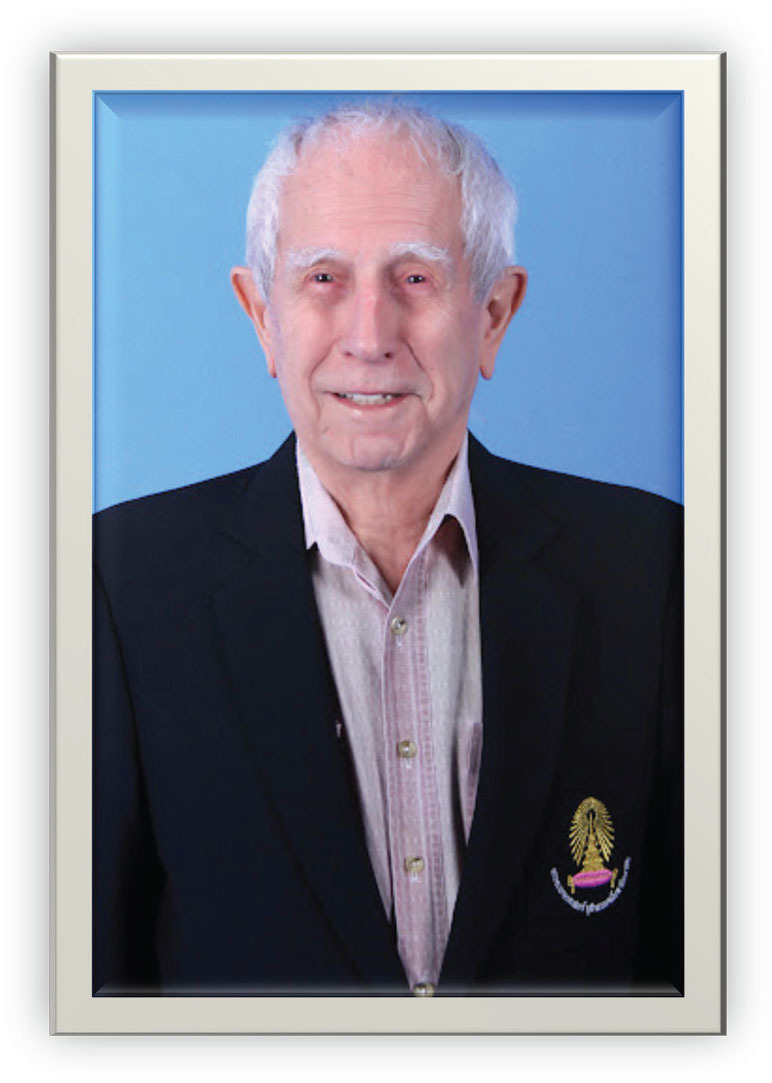


Born in Prague in 1927, during the Second World War, Professor Henry Wilde had been imprisoned by the Gestapo when he was 17 years old, as a suspected member of the Czech resistance movement. In 1945, after the Second World War, Professor Wilde settled in Alaska, in the United States, where he had his formal education and became a physician; he practiced in Alaska for many years.

Professor Wilde qualified as a general internist and infectious disease physician. He joined the US Government services in 1960 as a commissioned US foreign service officer. He has been assigned to many parts of the world, including West Africa, Viet Nam, Lebanon, Yugoslavia, and Thailand.

Professor Wilde retired from US Government service in 1984, with the rank of Minister-Counselor in the US Foreign Service. He became a fulltime faculty member of the Faculty of Medicine, at Chulalongkorn University, and an active member of the research team at the Thai Red Cross Society, where his work focused on tropical diseases research and control, including rabies control in humans and animals [1], [2], [3], [4], [5], [6], [7], [8], [9], [10], other infectious and neglected tropical diseases [11], [12], [13], [14], [15], [16], animal bites and toxins [17, 18], travel medicine [19], [20], [21], [22], ethics in clinical research and medical practices [23], [24], [25], [26], [27], [28], and medical education [29], [30], [31], among others.

Professor Wilde qualified as Diplomate in the American Board of Internal Medicine; Fellow of the American College of Physicians (FACP); Fellow of the Royal College of Tropical Medicine (UK); and a Member of the Medical Council of Thailand. Because of his credentials and being a good researcher and a clinician with an inquisitive mind, Professor Wilde served well as an excellent educator. He helped students, residents, and faculties in transforming clinical and research data into scientific publications. He was a Co-Editor of *Asian Biomedicine Journal*, which is currently indexed by PubMed Central. He had an active medical licensure in Alaska, England, and Thailand and had previously practiced in Canada, Washington DC, Lebanon, States of Washington, and Oregon. He held many positions, including Professor of Medicine, at Chulalongkorn University, and as a member of the World Health Organization (WHO) Collaborating Centre for Research and Training in Viral Zoonoses, at the Thai Red Cross Society. He served as a member of several WHO committees. He had about 200 publications in scientific peer-review journals. His awards and decorations include the National Resistance Medal, Czech Republic (for service as messenger during World War II); Senior Service Awards, US Foreign Service, 1971 and 1983; Commander, Order of White Elephant, Thailand, 1960; Knight Commander of the Crown, Thailand, 1995; and Lifetime Achievement Award for leadership and outstanding contributions in rabies control, India, 2012.

Professor Wilde passed away on June 6, 2024. We would like to convey our heartfelt gratitude to him, who worked tirelessly to make the world a better place for individuals and organizations. His ideas, ideals, and friendships had served as a tremendous example of a tireless colleague who tried to make science the cornerstone of education, ethical standards, disease prevention, and control.
